# Analysis of *Salmonella enterica* Serovar Typhi by Outer Membrane Protein (OMP) Profiling, Random Amplification of Polymorphic DNA (RAPD) and Pulsed Field Gel Electrophoresis (PFGE)

**DOI:** 10.21315/tlsr2019.30.1.4

**Published:** 2019-01-31

**Authors:** Yashwant Kumar, Kavaratty Raju Mani, Ajay Kumar Tahlan

**Affiliations:** National *Salmonella* and *Escherichia* Centre, Central Research Institute, Kasauli—173204, Himachal Pradesh, India

**Keywords:** *Salmonella* Typhi, Analysis, Pulsed Field Gel Electrophoresis (PFGE), Outer Membrane Protein (OMP), Random Amplification of Polymorphic DNA (RAPD)

## Abstract

A number of countries, including developed countries, still have typhoid fever as a major problem resulting in frequent outbreaks. The importance of controlling spread of typhoid fever is well known and necessitates periodic studies to delineate epidemiological relationships. Although phage typing remains to be the preferred conventional method for characterisation of typhoid bacilli, it is of limited use due to prevalence of few predominant phage types in the country like India. Therefore, an effort has been made to assess three molecular methods [Outer Membrane Protein (OMP) Profiling, Random Amplification of Polymorphic DNA (RAPD) and Pulsed Field Gel Electrophoresis (PFGE)] for typing of *Salmonella enterica* serovar Typhi. 128 *Salmonella enterica* serovar Typhi isolates were identified using biotyping and serotyping followed by antimicrobial susceptibility testing. These isolates were further subjected to OMP analysis, RAPD and PFGE. PFGE (114 unique clusters) was found to be the most discriminatory method followed by RAPD (94 unique clusters) and OMP profiling (50 unique clusters). Multidrug resistant strains were well discriminated by all three methods used in the study. PFGE still remains the most preferred method for detailed epidemiological investigations. However, random amplification of polymorphic DNA and outer membrane protein profiling can also be considered for molecular discrimination of the isolates in the laboratories lacking high-end facilities.

## INTRODUCTION

Salmonella serovars are responsible for infections occuring in developing as well as developed world and have been a major concern in terms of economic burden due to high morbidity (Obaro *et al*. 2017). Among them, *Salmonella enterica* serovar Typhi leads to infections in children and adults resulting in high morbidity (Paul & Bandyopadhyay 2017). Globally, annual number of cases of typhoid fever is estimated to be 9.9 to 24.2 million comprising high proportion from Indian subcontinent (Antillón *et al*. 2017). Moreover, despite the availability of effective vaccine and antimicrobials, typhoid fever still remains a significant health problem and posing challenges to health authorities due to the ever increasing resistance to various classes of antimicrobials leading to limited therapeutic options (Khanal *et al*. 2017).

Epidemiological investigation of *Salmonella enterica* serovar Typhi becomes relevant, primarily in the endemic areas which will facilitate delineation of distribution and transmission patterns of the strains and help health authorities to revise and establish effective prophylactic and therapeutic strategies.

Various conventional phenotypic methods comprising biotyping, serotyping, phage typing and antimicrobial susceptibility testing, are being used frequently for epidemiological investigations. Although these methods lay down the basis of phenotypic characterisation of the isolates, these are unable to provide enough epidemiological information. Moreover, serotyping, being the most widely used phenotypic method, fails to provide appropriate information due to complex serotyping scheme and lack of comparison among different laboratories thereby limiting its application to the reference laboratories only (Parmley *et al*. 2013).

Therefore, various novel genotyping methods have been developed and utilised (Martínez-Gamboa *et al*. 2015; Sankar *et al*. 2013; Tau *et al*. 2017; Silva *et al*. 2017) to delineate epidemiological relationships among various isolates even within the same phage types (Baggesen *et al*. 2010; Barco *et al*. 2013). But the search still continues for the easy to use efficient method capable of differentiating strains of similar phenotype (Yachison *et al*. 2017). Comparison of various characterisation methods available for *Salmonella enterica* serovar Typhi is given in Table 1.

Hence, we evaluated and compared one phenotypic method (OMP profiling) and two genotypic methods (RAPD and PFGE) for their potential to distinguish among various strains of *Salmonella enterica* serovar Typhi received at National Salmonella and Escherichia Centre from different parts of India.

## MATERIALS AND METHODS

### Bacterial Strains

One hundred and twenty eight (128) biochemically and serologically characterised isolates stored at National Salmonella and Escherichia Centre constituted material for the study. These isolates were received at the centre from different states of India viz., Maharastra (64.84%), Tamil Nadu (19.53%), Karnataka (7.03%), Gujrat (5.47%), Madhya Pradesh (2.34%) and Uttrakhand (0.78%) The bacteria were isolated from human blood (96.1%), human faeces (1.56%), waste water (1.56%) and buffalo meat (0.78%).

### Biochemical Identification and Serotyping

The isolates were subjected to biochemical identification as described by Holt *et al*. (1994) by using a battery of various biochemical media (Hi Media Laboratories, Pvt. Ltd., Mumbai, India). The isolates were further subjected to serotyping as described by Hendriksen and Larsen (2004) by using various ‘O’ and ‘H’ polyvalent and factor antisera (Becton Dickinson, USA; Denka Seiken Laboratories, Tokyo, Japan; Statens Serum Institute, Denmark). Briefly, the isolate was inoculated on nutrient agar and swarm agar for ‘O’ antigen and ‘H’ antigen typing respectively and incubated overnight at 37°C. The growth was emulsified in sterile normal saline on glass slide and then mixed with polyvalent and monovalent antisera to determine the serotype on the basis of agglutination. Auto-agglutinating strains were designated as rough. The results were interpreted using Kauffman and White scheme (Grimont & Weill 2007).

### Antimicrobial Susceptibility Testing

Antimicrobial susceptibility pattern of the isolates were determined by Kirby-Bauer disk diffusion method as per CLSI guidelines (Clinical and Laboratory Standards Institute [CLSI] 2007). The antibiotic discs (Hi Media Lab. Pvt. Ltd., Mumbai, India) used in the study were cefotaxime (30 μg), ceftazidime (30 μg), ciprofloxacin (5 μg), tetracycline (30 μg), chloramphenicol (30 μg), streptomycin (10 μg) ampicillin (10 μg), gentamicin (10 μg), kanamycin (30 μg), nalidixic acid (30 μg) and trimethoprim (5 μg).

### Outer Membrane Proteins Profiling

Extraction of outer membrane proteins was done as described by Hamid and Jain (2008). Briefly, the bacterial mass was harvested and washed thrice using Tris-HCl (10 mM, pH 7.5). The bacteria (1.0 g wet weight) was extracted using 20 ml of extraction buffer (10 mM Tris-HCl, pH 7.5, 10 mM EDTA containing 6 M urea) at 4°C for 1 h. The extract was further subjected to dialysis using distilled water with frequent changes for three days. The material obtained after dialysis was centrifuged at 6,000 rpm at 4°C for 1 h and the supernatant was collected. The protein mixture was further separated into its constituents by SDS-PAGE employing 12% separating gel as described by Ausubel *et al*. (2003). Briefly, SDS PAGE gel was cast using 3.9% stacking gel and 12% separating gel in the SDS-PAGE electrophoresis unit (Bangalore Genie, Bangalore, India). The protein mixture was heated at 100°C for 5 min in 0.05 M of Tris-HC1 buffer (2.5% SDS, 5% 2-mercaptoethanol, 25% glycerol, and 0.03% bromophenol blue). The samples were loaded in the wells and subjected to electrophoresis at 15 mA using 1X SDS electrophoresis buffer. After completion, the gel was stained with 0.25% Coomassie brilliant blue (Sigma-Aldrich, USA) and followed by imaging using Gel Doc XR+ System (Bio-Rad Laboratories, Hercules, USA).

### RAPD (Random Amplification of Polymorphic DNA)

Bacterial DNA was extracted as described by Ausubel *et al*. (2003). Extracted DNA was further subjected to RAPD using a PCR buffer system containing Tris-HCl (10 mM, pH 9.0), KCl (50 mM), MgCl_2_ (2.5 mM), gelatin (0.01%), Triton X-100 (0.1%), 0.2 mM of each of the deoxynucleotide triphosphates, primer (50 pmol), and *Taq* polymerase (0.2 U) (Fermentas International Inc., Canada). PCR reaction was put up in a thermocycler (Mastercycler, Eppendorf, New York, USA) using pre-denaturation step at 94°C for 4 min followed by 35 cycles of 94°C for 1 min, 25°C for 1 min and 74°C for 2 min. The primer used to differentiate *Salmonella enterica serovar*. Typhi strains was RAPD1 (5′-GGTTGGGTGAGAATTGCACG-3′) (Van Belkum *et al*. 1994). Electrophoresis (100 mA) was done in 1.5% agarose gel to separate the PCR products. Gels were stained after electrophoresis using ethidium bromide (10 mg/ml) and photographed using gel documentation system (Gel Doc XR+ System, Bio-Rad Laboratories, Hercules, USA).

### PFGE (Pulsed Field Gel Electrophoresis)

PFGE of the bacterial isolates was performed using one-day standardised protocol of Pulsenet as described by Ribot *et al*. (2006). Briefly, bacteria were inoculated in Trypticase Soy Agar with 5% defibrinated sheep blood. The growth was then suspended in cell suspension buffer (100 mM EDTA,100 mM Tris, pH 8.0) followed by adjustment of cell density to 0.8–0.1 at 610 nm. Twenty microliters of proteinase K (20 mg/ml) was added to 200 μl of cell suspensions and mixed with of 1% SeaKem Gold agarose (Lonza, Basel, Switzerland). Agarose mixture (300 μl) was dispensed into plug molds (Bio-Rad Laboratories, Hercules, USA) and agarose was allowed to solidify. Agarose plugs were then transferred to lysis buffer (50 mM Tris, 50 mM EDTA, 1% Sarkosyl [pH 8.0]) containing 25 μl of proteinase K (20 mg/ml) and incubated for 2 h in shaking incubator (150–175 rpm at 54°C). Plugs were subjected to 15 min washing with sterile ultrapure water followed by 15 min washing with four times in shaking incubator. Agarose plugs containing DNA were cut into small pieces (2 mm) and subjected to digestion with XbaI (50U) at 37°C for 2 h. The comb was then loaded with the agarose plugs and electrophoresed in 1% SeaKem agarose gel prepared in 0.5X Tris-EDTA buffer using CHEF DRII (Bio-Rad Laboratories, Hercules, USA) with the following settings: switch times of 2.12 to 63.8 s at 6 V/cm for 18 h at 14°C. Bands were visualised using Gel Doc XR+ System (Bio-Rad Laboratories, Hercules, USA) after staining with ethidium bromide (1 mg/ml).

## DATA ANALYSIS

The banding patterns obtained from OMP profiling, RAPD and PFGE were analysed using Bionumerics 6.5 software package (Applied Maths, NV, Belgium). The software was used for preparation of dendrograms showing similarity index on the basis of banding patterns obtained during analysis of samples by different methods.

## RESULTS

**Figure 1:**
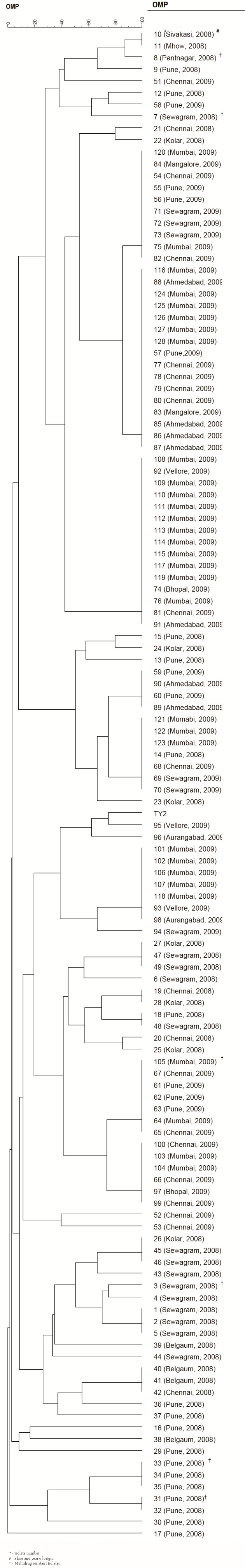
Analysis of the outer membrane protein profiles resulted in the dendrogram clustering 128 *Salmonella enterica* serovar Typhi into 50 distinct groups showing lesser discrimination ability than that in RAPD and PFGE. All six multidrug resistant isolates belonged to the different groups.

**Figure 2:**
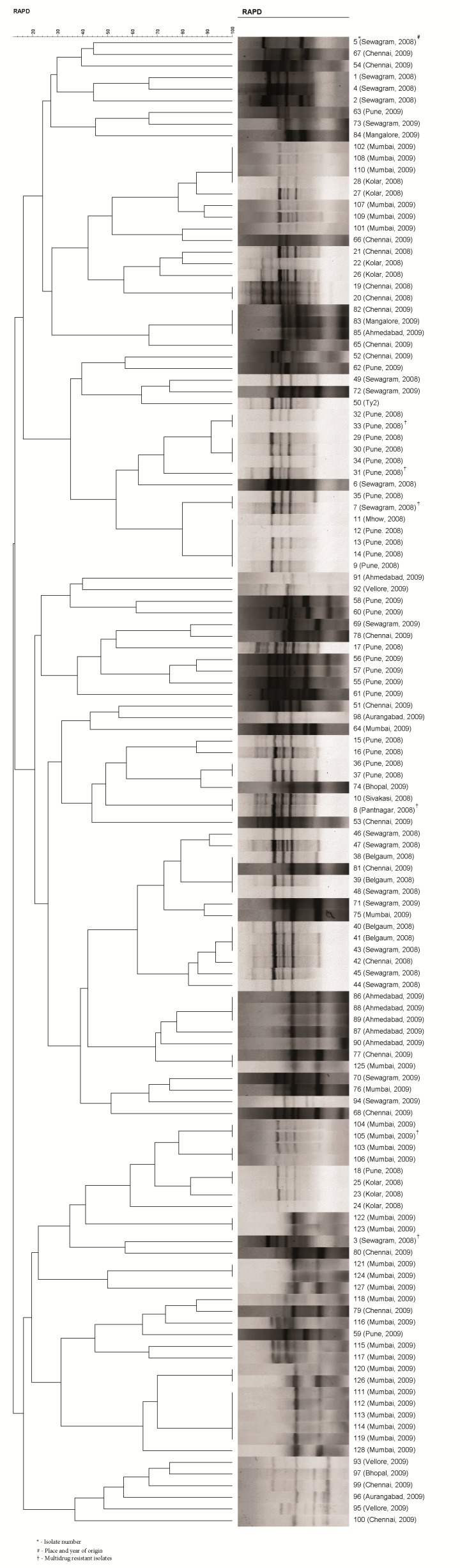
Dendrogram showing various RAPD patterns exhibits clustering of 128 *Salmonella enterica* serovar Typhi isolates into 94 distinct groups with larger number of indistinguishable isolates as compared to that in PFGE. These indistinguishable isolates are distributed among 20 groups containing 2–5 isolates. All multidrug resistant isolates belong to distinct groups.

**Figure 3:**
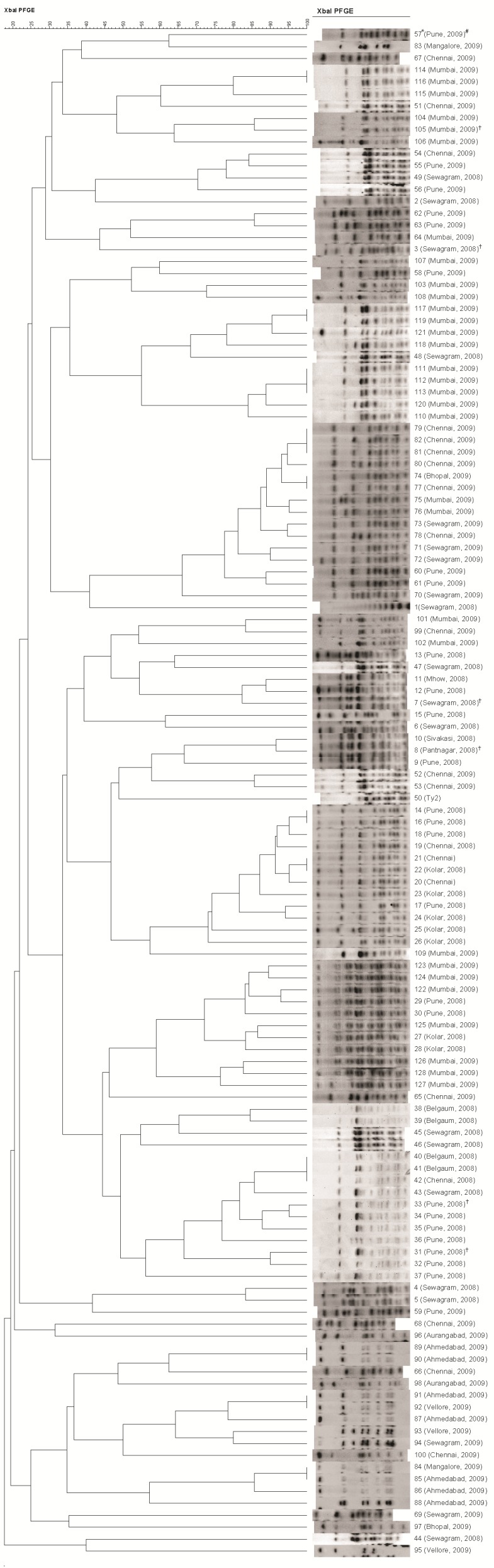
No clonal relationship was found among the multidrug resistant isolates (3, 7, 8, 31, 33 and 105) and all six of them belonged to distinct groups. 100% similarity was observed among 11 groups comprising isolate numbers - (114,116), (117,119), (111,112,113), (79.81,82), (74,77), (14,16), (21,22), (40,41,42), (89,90), (91,92) and (85,85). Among all indistinguishable groups, isolates in each group belong to the same place and year of origin except the groups comprising the isolates (41,42,43), (91,92) and (84,85) which were found to have different place of origin.

Among 128 isolates, only six isolates were found to be multidrug resistant with similar resistotype i.e., C-A-S-Na-Tr (Chloramphenicol, Ampicillin, Streptomycin, Nalidixic acid, Trimethoprim). When 128 isolates, received from various parts of India, were analysed using OMP profiling, RAPD and PFGE, outer membrane protein profiling was able to cluster 128 strains of *Salmonella enterica* serovar Typhi into 50 unique groups (Fig. 1) whereas RAPD and PFGE were able to group them into 94 unique groups (Fig. 2) and 114 unique groups (Fig. 3) respectively. The standard strain (Ty2) was well discriminated and comprised a distinct group by all the three methods (Figs. 1, 2 and 3). Similarly, all multidrug resistant isolates were also well discriminated by all the three methods (Table 2).

## DISCUSSION

Typhoid is endemic in India with significant amount of morbidity and mortality thereby necessitating its epidemiological monitoring. Moreover, emergence and spread of antimicrobial resistance among *Salmonella enterica* serovar Typhi also emphasise the need for epidemiological analysis of multidrug resistant strains. Policy makers in several developing countries have indicated the need of updated epidemiological data related to the incidence of typhoid fever in their countries before they incorporate the vaccines in routine immunisation programmes.

Although phenotypic methods are widely used worldwide, genotypic methods are more useful for thorough epidemiological analysis, utilising the evolutionary differences in DNA patterns. Therefore, in the present study two genotypic methods (RAPD and PFGE) were assessed for their epidemiological usefulness in addition to one phenotypic method (OMP profiling).

Outer membrane protein profiling has been considered by several authors for epidemiological analysis (Marhual *et al*. 2012; Jain *et al*. 2005; Munir *et al*. 2007) of various microorganisms. In the present study, considerable amount of heterogeneity (50 unique groups) was found among different isolates during analysis (Fig. 1) of the outer membrane protein profiles. But the discriminatory ability was found to be less than RAPD and PFGE (Fig. 2 and Fig. 3). Although OMP profiling was found to be less discriminative as compared to other two methods used in the study, it may be used as an epidemiological tool for preliminary discrimination in the laboratories where high end facilities are not available. Moreover, this method discriminated all multidrug resistant strains well (Table 2) thereby highlighting its possible usefulness for epidemiological analysis of *Salmonella enterica* serovar Typhi.

RAPD assay is considered to be useful due to its capability to generate polymorphisms and being simpler, economical and fast than most other molecular typing methods (Albufera *et al*. 2009; Bhowmick *et al*. 2012). In the present study, RAPD was able to discriminate among all multidrug resistant isolates (Fig. 2, Table 2) and exhibited its capability as a promising tool for epidemiological and characterisation studies. Similar findings have also been reported earlier by Nath *et al*. (2010), Jegadeeshkumar *et al*. (2010) and Shabarinath *et al*. (2007). RAPD was found to be more discriminative than OMP profiling and can be used by researchers for better differentiation among the strains in the laboratories lacking PFGE instrumentation. Moreover, the usability of RAPD for epidemiological analysis of *Salmonella enterica* serovar Typhi strains may also be increased by using more than one set of primers. However, RAPD being the PCR based method only rely on the amplicons of limited number of target DNA sites (Millemann *et al*. 2000) and is not suitable for differentiation of strains at clone level.

Pulsed field gel electrophoresis clustered 128 salmonella isolates into 114 groups based on the banding pattern similarity index (Fig. 3) and found to be the most discriminating method among all the three methods used in the present study. Dendrogram of PFGE exhibited clonal relationships in 11 groups. Among these, four groups (P32, P62, P86 and P108) exhibited clonal relationships among isolates from different places of the country (Table 3) showing possible dissemination of *Salmonella enterica* serovar Typhi clones from one geographical area to another (Fig. 3). Two groups (P100 and P103) were found to have clonal relationships among the isolates from different sources (Table 3), which depicts possible spread of the clone from one reservoir to another in the same geographic region. This emphasises the usefulness of PFGE to trace out the source of infection during epidemiological investigations of outbreaks. As observed in OMP profiling and RAPD, all multidrug resistant isolates belonged to different PFGE groups and shows that these multidrug resistant strains have no clonal relationship and may have originated and spread independently (Table 2, Fig. 3).

PFGE typing has been reported as an important molecular technique for epidemiological analysis of many bacterial pathogens including *Salmonellae* (Woo 2005) and employed for the epidemiological analysis of various salmonella serovars (Fendri *et al*. 2013; Deekshit *et al*. 2016). Our study also exhibited PFGE as the most discriminative method and therefore may be used as a method of choice for epidemiological studies. PFGE has emerged as an important epidemiological tool and possess advantage of discriminating on the basis of whole genome digested with restriction enzymes. Although PFGE has the highest discriminatory ability, a separate pulsotype will only be generated if the DNA variation is able to affect the size and thereby electrophoretic mobility of the band. Use of secondary restriction enzyme can however overcome this limitation and also lead to increase in discriminatory potential (Fernandez *et al*. 2003). Due to generation of large number of bands, PFGE also result in more accurate cluster analysis when variations of two or three bands occur, leading to high similarity index among closely related strains. Moreover, use of a uniform, well standardised and validated protocol, as provided by PulseNet, is indispensable in order to have meaningful comparative analysis of PFGE patterns from different epidemiological studies among various laboratories throughout the world.

## CONCLUSION

Considering the present scenario commanded by the emergence and spread of multidrug resistance among *Salmonella enterica* serovar Typhi in addition to high morbidity and mortality associated with typhoid, continuous surveillance using various epidemiological tools is required. PFGE should be used as a potential epidemiological tool due to its high degree of discrimination ability. However, considering the cost and time involved in case of PFGE, RAPD and OMP profiling should be considered for preliminary differentiation of strains at the level of regional laboratories lacking instrumentation required for PFGE. Health authorities should also develop and execute strong and effective surveillance strategies ensuring timely submission of the isolates to the designated laboratories and compilation of the data generated by different countries so as to prepare database of different *Salmonella enterica* serovar Typhi strains prevalent globally. Data on antimicrobial profile of these strains should also be generated and compiled to assess and monitor the level of antimicrobial resistance prevelant among different strains of *Salmonella enterica* serovar Typhi. Moreover, these laboratories should be equipped with PFGE equipment besides being collaborated to global surveillance networks such as Salm-Surv and PulseNet.

## Figures and Tables

**Table 1:** Comparison of different methods employed for typing of *Salmonella* Typhi.

Parameter	Testing Methods							

	Plasmid profiling	Phage typing	Ribotyping	IS200 Typing	MLST	OMP Profiling	RAPD	PFGE
Cost	Low	Moderate	Moderate	Moderate	High	Low	Moderate	High
Run time	3–5 h	18–24 h	5–7 h	3–5 h	18–24 h	3–5 h	3–5 h	20–24 h
Training	Minimal	Moderate	High	Minimal	High	Minimal	Minimal	High
Difficulty level	Low	Moderate	High	Low	High	Low	Low	High
Instrumentation	Simple	Simple	High-end	Moderate	High-end	Simple	Moderate	High-end

**Table 2:** Distribution of multidrug resistant isolates among various groups.

Isolate no.	Source	Place and year of origin	OMP group	RAPD group	PFGE group
3	Blood	Sewagram (2008)	O36	R76	P18
7	Blood	Sewagram (2008)	O7	R31	P50
8	Blood	Pantnagar (2008)	O2	R51	P54
31	Blood	Pune (2008)	O48	R29	P92
33	Blood	Pune (2008)	O47	R27	P88
105	Blood	Mumbai (2009)	O30	R70	P8

**Table 3:** Clonal relationships among *Salmonella enterica* serovar Typhi isolates on the basis of PFGE.

Isolate no.	Source	Place of origin	Year of origin	PFGE group
114	Blood	Mumbai	2009	P4
116	Blood	Mumbai	2009	
117	Blood	Mumbai	2009	P23
119	Blood	Mumbai	2009	
111	Blood	Mumbai	2009	P27
112	Blood	Mumbai	2009	
113	Blood	Mumbai	2009	
79	Blood	Chennai	2009	P30
81	Blood	Chennai	2009	
82	Blood	Chennai	2009	
74	Blood	Bhopal	2009	P32
77	Blood	Chennai	2009	
14	Blood	Pune	2008	P59
16	Blood	Pune	2008	
21	Blood	Chennai	2008	P62
22	Blood	Kolar	2008	
40	Blood	Belgaum	2008	P86
41	Blood	Belgaum	2008	
42	Blood	Chennai	2008	
89	Feces	Ahmedabad	2009	P100
90	Blood	Ahmedabad	2009	
91	Waste water	Ahmedabad	2009	P103
92	Blood	Ahmedabad	2009	
84	Blood	Mangalore	2009	P108
85	Blood	Ahmedabad	2009	

## References

[b1-tlsr-30-1-57] Albufera U, Bhugaloo-Vial P, Issack M, Jaufeerally-Fakim Y (2009). Molecular characterization of Salmonella isolates by REP-PCR and RAPD analysis. Infection, Genetics and Evolution.

[b2-tlsr-30-1-57] Antillón M, Warren JL, Crawford FW, Weinberger DM, Kürüm E, Pak GD, Marks F, Pitzer VE (2017). The burden of typhoid fever in low- and middle-income countries: A meta-regression approach. PLoS Neglected Tropical Diseases.

[b3-tlsr-30-1-57] Ausubel FM (2003). Current protocols in molecular biology.

[b4-tlsr-30-1-57] Baggesen DL, Sørensen G, Nielsen EM, Wegener HC (2010). Phage typing of Salmonella Typhimurium: Is it still a useful tool for surveillance and outbreak investigation?. Eurourveillance.

[b5-tlsr-30-1-57] Barco L, Barrucci F, Olsen JE, Ricci A (2013). Salmonella source attribution based on microbial subtyping. International Journal of Food Microbiology.

[b6-tlsr-30-1-57] Bhowmick PP, Srikumar S, Devegowda D, Shekar M, Ruwandeepika HAD, Karunasagar I (2012). Serotyping & molecular characterization for study of genetic diversity among seafood associated nontyphoidal Salmonella serovars. Indian Journal of Medical Research.

[b7-tlsr-30-1-57] Clinical and Laboratory Standards Institute (CLSI) (2007). Performance standards for antimicrobial susceptibility testing, seventeenth informational supplement.

[b8-tlsr-30-1-57] Deekshit VK, Kumar BK, Rai P, Karunasagar I (2016). Antibiotic resistance and molecular characterization of seafood isolates of nontyphoidal Salmonella by PFGE. Procedia Food Science.

[b9-tlsr-30-1-57] Fendri I, Hassena AB, Grosset N, Barkallah M, Khannous L, Chuat V (2013). Genetic diversity of food-isolated Salmonella strains through pulsed field gel electrophoresis (PFGE) and enterobacterial repetitive intergenic consensus (ERIC-PCR). PLoS One.

[b10-tlsr-30-1-57] Fernandez J, Fica A, Ebensperger G, Calfullan H, Prat S, Fernandez A, Alexandre M, Heitmann I (2003). Analysis of molecular epidemiology of Chilean *Salmonella enterica* serotype Enteritidis isolates by pulsed-field gel electrophoresis and bacteriophage typing. Journal of Clinical Microbiology.

[b11-tlsr-30-1-57] Grimont PAD, Weill FX (2007). Antigenic formulae of the Salmonella serovars.

[b12-tlsr-30-1-57] Hamid N, Jain SK (2008). Characterization of an outer membrane protein of *Salmonella enterica* serovar Typhimurium that confers protection against typhoid. Clinical and Vaccine Immunology.

[b13-tlsr-30-1-57] Hendriksen RS, Larsen JN (2004). Laboratory protocols: Serotyping of Salmonella enterica O and H antigens.

[b14-tlsr-30-1-57] Holt JG, Krieg NR, Sneath PHA, Staley JT, Williams ST, Holt GJ (1994). Group 5: Facultatively anaerobic Gram negative rods. Bergey manual of determinative bacteriology.

[b15-tlsr-30-1-57] Jain A, Roy A, Rank DN, Joshi CG, Purohi JH (2005). Characterization of the *Pasteurella multocida* isolates by their outer membrane protein profiles. Indian Journal of Comparative Microbiology, Immunology and Infectious Diseases.

[b16-tlsr-30-1-57] Jegadeeshkumar D, Saritha V, Moorthy K, Suresh kumar BT (2010). Prevalence, antibiotic resistance and RAPD analysis of food isolates of Salmonella species. International Journal of Biological Technology.

[b17-tlsr-30-1-57] Khanal PR, Satyal D, Bhetwal A, Maharjan A, Shakya S, Tandukar S, Parajuli NP (2017). Renaissance of conventional first-line antibiotics in salmonella enterica clinical isolates: Assessment of MICs for therapeutic antimicrobials in enteric fever cases from Nepal. BioMed Research International.

[b18-tlsr-30-1-57] Marhual NP, Das BK, Pradhan J, Swain P, Mishra BK, Eknath AE (2012). RAPD-PCR and outer membrane protein characterization of *Vibrio alginolyticus* and *V. parahaemolyticus* isolated from diseased shrimp. Israeli Journal of Agriculture.

[b19-tlsr-30-1-57] Martínez-Gamboa A, Silva C, Fernández-Mora M, Wiesner M, Ponce de León A, Calva E (2015). IS200 and multilocus sequence typing for the identification of Salmonella enterica serovar Typhi strains from Indonesia. International Microbiology.

[b20-tlsr-30-1-57] Millemann Y, Gaubert S, Remy D, Colmin C (2000). Evaluation of IS*200*-PCR and comparison with other molecular markers to trace *Salmonella enterica* subsp. *enterica* serotype Typhimurium bovine isolates from farm to meat. Journal of Clinical Microbiology.

[b21-tlsr-30-1-57] Munir R, Shahwar D, Farooq U, Nawaz I, Shahzad I, Khanum A (2007). Outer membrane protein profiling of *Pasteurella multocida*. Pakistan Veterinary Journal.

[b22-tlsr-30-1-57] Nath G, Maurya P, Gulati AK (2010). ERIC PCR and RAPD based fingerprinting of *Salmonella* Typhi strains isolated over a period of two decades. Infection, Genetics and Evolution.

[b23-tlsr-30-1-57] Obaro SK, Iroh Tam PY, Mintz ED (2017). The unrecognized burden of typhoid fever. Expert Review of Vaccines.

[b24-tlsr-30-1-57] Parmley EJ, Pintar K, Majowicz S, Avery B, Cook A, Jokinen C, lapen DR, Topp E, Edge TA, Gilmour M, Pollari F, Reid-Smith R, Irwin R (2013). A Canadian application of one health: Integration of Salmonella data from various Canadian surveillance programs (2005–2010). Foodborne Pathogens Disease.

[b25-tlsr-30-1-57] Paul UK, Bandyopadhyay A (2017). Typhoid fever: A review. International Journal of Advanced Medicine.

[b26-tlsr-30-1-57] Ribot EM, Fair MA, Gautam R, Cameron DN, Hunter SB, Swaminathan B, Barrett TJ (2006). Standardization of pulsed-field gel electrophoresis protocols for the subtyping of *Escherichia coli* O157:H7, *Salmonella*, and *Shigella* for PulseNet. Foodborne Pathogens Disease.

[b27-tlsr-30-1-57] Sankar S, Kuppanan S, Nandagopal B, Sridharan G (2013). Diversity of Salmonella enterica serovar Typhi strains collected from india using variable number tandem repeat (VNTR)-PCR analysis. Molecular Diagnosis & Therapy.

[b28-tlsr-30-1-57] Shabarinath S, Sanath Kumar H, Khushiramani R, Karunasagar I, Karunasagar I (2007). Detection and characterization of Salmonella associated with tropical seafood. International Journal of Food Microbiology.

[b29-tlsr-30-1-57] Silva C, Betancor L, García C, Astocondor L, Hinostroza N, Bisio J, Rivera J, Perezgasga L, Escanda VP, Yim L, Jacobs J, Portillo FG, Chabalgoity JA, Puente JL, the Salmober CYTED Network (2017). Characterization of Salmonella enterica isolates causing bacteremia in Lima, Peru, using multiple typing methods. PLoS One.

[b30-tlsr-30-1-57] Tau NP, Smith AM, Wain JR, Tarupiwa A, Coulibaly KJ, Keddy KH, Germ-Sa (2017). Development and evaluation of a multiple-locus variable-number tandem-repeats analysis assay for subtyping Salmonella Typhi strains from sub-Saharan Africa. Journal of Medical Microbiology.

[b31-tlsr-30-1-57] Van Belkum A, Bax R, Prevost G (1994). Comparison of four genotyping assays for epidemiological study of methicillin resistant *Staphylococcus aureus*. European Journal of Clinical Microbiology & Infectious Diseases.

[b32-tlsr-30-1-57] Woo YK (2005). Finding the sources of Korean Salmonella enterica serovar Enteritidis PT 4 isolates by pulsed-field gel electrophoresis. Journal of Microbiology.

[b33-tlsr-30-1-57] Yachison CA, Yoshida C, Robertson J, Nash JHE, Kruczkiewicz P, Taboada EN, Walker M, Reimer A, Christianson S, Nichani A, Nadon C, The PulseNet Canada Steering Committee (2017). The validation and implications of using whole genome sequencing as a replacement for traditional serotyping for a national salmonella reference laboratory. Frontiers in Microbiology.

